# Improved Transductive Support Vector Machine for a Small Labelled Set in Motor Imagery-Based Brain-Computer Interface

**DOI:** 10.1155/2019/2087132

**Published:** 2019-11-25

**Authors:** Yilu Xu, Jing Hua, Hua Zhang, Ronghua Hu, Xin Huang, Jizhong Liu, Fumin Guo

**Affiliations:** ^1^School of Mechatronics Engineering, Nanchang University, Nanchang 330031, China; ^2^School of Software, Jiangxi Agricultural University, Nanchang 330045, China; ^3^Department of Computer Science and Technology, Tongji University, Shanghai 201804, China

## Abstract

Long and tedious calibration time hinders the development of motor imagery- (MI-) based brain-computer interface (BCI). To tackle this problem, we use a limited labelled set and a relatively large unlabelled set from the same subject for training based on the transductive support vector machine (TSVM) framework. We first introduce an improved TSVM (ITSVM) method, in which a comprehensive feature of each sample consists of its common spatial patterns (CSP) feature and its geometric feature. Moreover, we use the concave-convex procedure (CCCP) to solve the optimization problem of TSVM under a new balancing constraint that can address the unknown distribution of the unlabelled set by considering various possible distributions. In addition, we propose an improved self-training TSVM (IST-TSVM) method that can iteratively perform CSP feature extraction and ITSVM classification using an expanded labelled set. Extensive experimental results on dataset IV-a from BCI competition III and dataset II-a from BCI competition IV show that our algorithms outperform the other competing algorithms, where the sizes and distributions of the labelled sets are variable. In particular, IST-TSVM provides average accuracies of 63.25% and 69.43% with the abovementioned two datasets, respectively, where only four positive labelled samples and sixteen negative labelled samples are used. Therefore, our algorithms can provide an alternative way to reduce the calibration time.

## 1. Introduction

A brain-computer interface (BCI) system can allow people to communicate directly with electronic equipment using their brain activity and without using their peripheral nerves and muscles [1]. In a noninvasive BCI system, electroencephalogram (EEG) signals are used to measure brain activity due to their safety and convenience [2]. In this paper, we focus on EEG signals of motor imagery (MI), which are invoked by either real or imagined movements of feet, hands, or tongue [3]. An MI-based BCI system is suitable for use in military, entertainment, and rehabilitation engineering systems.

However, due to the inherent nonstationarity of EEG signals, long and tedious calibration time is one of the key issues preventing broad use of MI-based BCI [4, 5]. Reducing the calibration time without loss of accuracy is a major challenge. To solve this problem, semisupervised learning (SSL) classifiers can use a small labelled set and a relatively large unlabelled set from the same subject for training.

In general, SSL classifiers can be categorized into generative, self-training, cotraining, graph-based, and transductive support vector machine (TSVM) models. A generative model iteratively uses the expectation maximization (EM) technique to build a probabilistic model with the aid of labelled and unlabelled data. Nevertheless, a generative model emphasizes that the labelled data must follow the Gaussian distribution [6]. A self-training model selects a supervised learning classifier as the base learner, which is retrained continually using the initial labelled data and the unlabelled data with high confidence [7–10]. Likewise, in the cotraining model, two supervised learning classifiers are iteratively trained using the other classifier's previous classification results [11, 12]. The accuracies of the self-training and cotraining models decrease when the unlabelled data are assigned incorrect labels. A graph-based model constructs a weighted graph to explore the manifold structure behind the labelled and unlabelled data [13–16]. However, it is difficult to develop a good graph in general situations. The TSVM model learns the decision boundary going through low-density regions and maximizes the margin between different clusters using the labelled and unlabelled data [17]. Nevertheless, the TSVM model may converge to a local optimum because of the nonconvex optimization problem. Thus, each SSL model has clear disadvantages.

In a BCI system, support vector machine (SVM) has been commonly used with small, nonlinear, high-dimensional EEG-labelled sets [7, 8]. Therefore, we pay more attention to the TSVM model, which originated from SVM [18, 19]. TSVM-light was an early implementation of the TSVM model, which was used to determine the maximum margin by switching different labels for a pair of unlabelled data during each iteration [18]. However, there is a nonconvex optimization problem in TSVM-light due to the nondifferentiability of the Hinge loss function on the unlabelled samples. To tackle this drawback, concave-convex procedure (CCCP) was used to decompose the optimization problem into its concave and convex parts [20]. However, CCCP could not scale well with larger datasets. Robust TSVM (RTSVM) provided higher computational efficiency for millions of samples by using the stochastic gradient (SG) method to solve the primal optimization problem [21]. Due to insufficient domain knowledge, it remains challenging for the TSVM model to provide high accuracy when used with obscure unlabelled data [22]. Based on manifold assumption, the graph-based model can be used with a large unlabelled set to describe the global distribution of the data. Recently, many graph-based semisupervised SVM (S3VM) classifiers were studied extensively in the literature, such as spatial-spectral label propagation based on SVM (SS-LPSVM) [23] and TSVM based on active learning (AL) and graph (TSVM_AL+graph_) [24]. SS-LPSVM and TSVM_AL+graph_ formulated information on the manifold structure using the Laplacian regularization term, which was added to the objective function in SVM. However, it was difficult to determine the optimal parameters using cross validation under the condition of small labelled sets. Consequently, such important parameters were always defined empirically. Semisupervised classification with low-density separation (LDS) was used to transform the original features of all samples into the geometric features [25]. Despite this advancement, the transformation procedure may omit important original information.

Moreover, it may be unreasonable to preset the ratio of positive to negative samples in the unlabelled set to be equal to the ratio in the labelled set in many TSVM methods [20, 21, 24, 25], especially when the small labelled set is extremely unbalanced. Incorrect estimation of this ratio may decrease the classification accuracy. To address this problem, Zhang designed a robust S3VM method via ensemble learning, where various distributions of the unlabelled set were considered [26].

Feature learning is as important as classifier learning in a BCI system. The common spatial patterns (CSP) method is commonly used with EEG signals because CSP can provide efficient feature extraction and dimension reduction [27, 28]. However, CSP is a supervised feature learning method. A limited labelled set may result in an unreliable CSP transformation matrix, which can directly affect the accuracy of feature vectors in all samples and consequently decrease the classification accuracy. To solve this problem, Li introduced an S3VM method based on the self-training model, in which feature learning and classifier learning were performed jointly and iteratively. In this method, the CSP transformation matrix and SVM classifier were successively updated by exploiting the initial labelled data and all or part of the unlabelled data with new labels learned during the previous iteration [7]. Similarly, many self-training and cotraining methods classify EEG signals using different supervised algorithms as the base learners, such as linear discriminant analysis (LDA), Bayesian LDA (BLDA), biomimetic pattern recognition (BPR), or sparse representation (SR) [8–12].

Motivated by the aforementioned studies, we formulate an improved TSVM (ITSVM) method by combining the TSVM model with a graph-based model. In this method, we construct the variation of a weighted graph as proposed by Chapelle [25] in order to explore the potential distribution of all samples in a semisupervised way. Then, we introduce a comprehensive feature for each sample, which consists of its CSP feature and its geometric feature. In addition, we use CCCP to solve the nonconvex optimization problem. Inspired by Zhang [26], in order to determine the unknown distribution of the unlabelled set, we impose a new balancing constraint that considers various possible distributions of the unlabelled set. As mentioned above, feature learning is critical for the BCI system. Thus, we develop an improved self-training TSVM (IST-TSVM) method that can execute CSP and our proposed ITSVM method jointly and iteratively. The contributions of our work are summarized as follows:

1. We propose an ITSVM method that can maximize the margin between different clusters and provide different views of all samples based on their CSP and geometric features.
2. In contrast to the traditional definition, we impose a new balancing constraint on the optimization problem in TSVM to address the unknown distribution of the unlabelled set.
3. Most existing self-training methods adopt supervised methods as the base learners. Here, we present an IST-TSVM method based on our confidence criterion and semisupervised ITSVM approach to utilize the unlabelled data in feature and classifier learning.
4. We performed extensive experiments to evaluate the efficiency of our proposed algorithms using small labelled sets with balanced or unbalanced classes. In particular, IST-TSVM outperforms the competing TSVM methods when used with extremely unbalanced labelled sets.

The remainder of this paper is structured as follows. In Section 2, the TSVM model is briefly reviewed and the details of our two improved TSVM methods are described. The effectiveness of our proposed methods, using two famous MI-based BCI competition datasets, is evaluated in Section 3. A discussion of the experimental results is presented in Section 4. Finally, our conclusions are drawn in Section 5.

## 2. Methods

### 2.1. TSVM Model

Consider a dataset with *L* labelled samples *x*_*i*_ (1 ≤ *i* ≤ *L*) and *U* unlabelled samples *x*_*i*_ (*L*+1 ≤ *i* ≤ *L*+*U*). The labelled samples are initially assigned binary class labels *y*_*i* _(*y*_*i* _ ∈ {−1,  +1}).

TSVM aims to identify the optimal hyperplane that separates the labelled and unlabelled samples with maximum margin. The linear hyperplane can be characterized by *θ*=(*w*, *b*), where *w* is the normal of the hyperplane and *b* is a bias term. Compared with SVM, TSVM minimizes the cost function *J*(*θ*) by adding an “effect term” *C*_2_ *ε*_*i*_ for each unlabelled sample as follows [18]:

(1) $$\begin{array}{c} \arg\underset{\theta }{\min}\left(J\left(\theta \right)\right)=\arg\underset{\theta }{\min}\left(\frac{1}{2}{\left\|w\right\|}^{2}+C_{1}\overset{L}{\underset{i=1}{\sum }}\varepsilon_{i}+C_{2}\overset{L+U}{\underset{i=L+1}{\sum }}\varepsilon_{i}\right),\\ \begin{array}{cc} \text{subject to:} & \forall_{i=1}^{L}:y_{i}\left(wx_{i}+b\right)\geq 1-\varepsilon_{i},\left(\varepsilon_{i}\geq 0\right),\\ & \forall_{i=L+1}^{L+U}:\left|wx_{i}+b\right|\geq 1-\varepsilon_{i},\left(\varepsilon_{i}\geq 0\right), \end{array} \end{array}$$

where *C*_1_ (*C*_2_) is a user-specified parameter that can punish misclassified labelled or unlabelled samples. The slack variables *ε*_*i*_ are defined to handle inseparable data. Equation (1) can be rewritten as an unconstrained minimization problem:

(2) $$\begin{array}{c} \arg\underset{\theta }{\min}\left(J\left(\theta \right)\right)=\arg\underset{\theta }{\min}\left(\frac{1}{2}{\left\|w\right\|}^{2}+C_{1}\overset{L}{\underset{i=1}{\sum }}F\left(\;y_{i}\left(wx_{i}+b\right)\right)+C_{2}\overset{L+U}{\underset{i=L+1}{\sum }}F\left(\left|wx_{i}+b\right|\right)\right), \end{array}$$

where *F*(·) is the loss function. Earlier implementations of the TSVM model adopted different loss functions. TSVM-light used the classical Hinge loss function *H*_1_(*t*)= max(0,  1 − *t*). However, the nondifferentiability of *H*_1_(|*t*|) on the unlabelled samples produces a nonconvex optimization problem. Thus, *H*_1_(|*t*|) was replaced by exp(−3*t* ^2^) in the LDS method [25]. However, most TSVM methods employed a symmetric ramp loss function for unlabelled samples [20, 21].

### 2.2. ITSVM Algorithm

**Figure 1:**
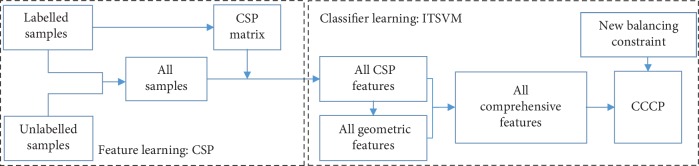
Signal processing flow chart for EEG signals.

Classification of EEG signals is a difficult task, especially when the labelled samples are sparse and unbalanced. In this paper, we first propose an ITSVM method that involves two stages. Specifically, in the first stage, we generate the comprehensive features for all samples based on their CSP features and geometric features to provide different views of the data. In the second stage, we use CCCP to solve the nonconvex loss function and define a new balancing constraint to adapt to the unknown distribution of the unlabelled set.  Figure 1 shows a flowchart illustrating the signal processing of the EEG signals, where CSP and ITSVM are successively employed for feature learning and classifier learning.

#### 2.2.1. Comprehensive Feature

CSP is an effective feature extraction method in a BCI system. Given an initial labelled set *D*_tr_={(*e*_1_,  *y*_1_),…, (*e*_*L*_,  *y*_*L*_)} and the remaining unlabelled set *D*_te_={*e*_*L*+1_,…, *e*_*L*+*U*_}, *e*_*i*_ is the *i*th EEG sample that was already preprocessed. As shown in Figure 1, CSP uses the labelled samples to calculate the CSP matrix, which can be used to maximize discrimination of the two classes of EEG signals. For a given CSP matrix *W*, the mapping of *e*_*i*_ is defined as the new time series *Z*=*We*_*i*_. Note that *W* consists of *m* pairs of spatial filters. Then, element *x*_*p*_^*i*^ in the CSP feature vector *x*_*i*_ for *e*_*i*_ is defined as follows:

(3) $$\begin{array}{c} x_{p}^{i}=\log\left({\mathrm{var}}_{p}^{i}\right),\;p=1,2,\ldots ,2m, \end{array}$$

where var_*p*_^*i*^ is the variance of the *p*th row of *Z*. Although CSP is robust against noise, a small labelled dataset may produce an unreliable CSP matrix, which directly influences the correctness of CSP features for all samples. Thus, it is valuable to explore the inherent spatial distribution with the assistance of unlabelled samples using graph-based SSL approaches.

In this paper, the original CSP features are converted into geometric features based on LDS [25]. LDS is used to build the nearest neighbour graph, and multidimensional scaling (MDS) is used to produce a new graphic representation of the data in a small number of dimensions [29]. In contrast to LDS, we replace the Euclidean distance with the cosine distance to measure the pairwise distance between two samples. The cosine distance can be used to correct inconsistencies in measurement standards that may be caused by high intersession variability among EEG signals. Moreover, it is assumed for LDS that two samples lying close to each other might belong to the same class. Then, LDS is used to calculate the shortest path between two samples in an unsupervised way. However, it is difficult to assess the classes of two samples if their shortest path has different classes of labelled samples. To overcome this problem, we build the nearest neighbour graph in a semisupervised way by maximizing the edge length between two labelled samples with different classes. The process for computing each geometric feature ${\tilde{x}}_{i}$ for the *i*th sample can be described as follows:

  Step 1: the pairwise distance between the *i*th and *j*th samples is initially weighted as follows:

(4) $$\begin{array}{c} d\left(x_{i},x_{j}\right)=\;1-\frac{x_{i}^{T}x_{j}}{\left\|x_{i}\right\|\left\|x_{j}\right\|},\;1\leq i,j\leq L+U, \end{array}$$

  where‖*x*_*i*_‖ and ‖*x*_*j*_ ‖are the lengths of *x*_*i*_ and *x*_*j*_, respectively. The last term in equation (4) is the cosine distance.
  Step 2: a fully connected graph with edge lengths *φ*(*x*_*i*_, *x*_*j*_) is constructed as follows:

(5) $$\begin{array}{c} \varphi \left(x_{i},x_{j}\right)=\exp\left(\rho d\left(x_{i},x_{j}\right)\right)-1\;,\;\rho =1. \end{array}$$

  Step 3: Before using *φ*(*x*_*i*_, *x*_*j*_) to compute the shortest path length *d*_sp_(*x*_*i*_, *x*_*j*_) based on Dijkstra's algorithm [30], we manually set *φ*(*x*_*i*1_, *x*_*j*1_)=max(*φ*(*x*_*i*_, *x*_*j*_)) (1 ≤ *i*1, *j*1 ≤ *L*,  1 ≤ *i*, *j* ≤ *L*+*U* and  *y*_*i*1 _ ≠ *y*_*j*1 _) . Therefore, it is impossible for labelled samples with different classes to exist along the shortest path.
  Step 4: the (*L*+*U*) × (*L*+*U*) matrix *G* of minimal squared *ρ*-path distances is defined as follows:

(6) $$\begin{array}{c} G_{ij}={\left(\frac{1}{\rho }\log\left(1+d_{\text{sp}}\left(x_{i},x_{j}\right)\right)\right)}^{2}. \end{array}$$

  Step 5: the positive eigenvalues *λ*_*i*_ and corresponding eigenvectors *V*_*i*_ of −*HGH*/2 are calculated using MDS, where *H*_*ij*_=*I*_*ij*_ − (1/(*L*+*U*)). *I*_*ij*_ is the element of identity matrix *I*. Both *H* and *I* are (*L*+*U*) × (*L*+*U*) matrices [29].
  Step 6: element ${\tilde{x}}_{ik}$ in the new graph-based representation ${\tilde{x}}_{i}$ is defined as

(7) $$\begin{array}{c} {\tilde{x}}_{ik}=V_{ik}\sqrt{\lambda_{k}},\;\;1\leq k\leq l, \end{array}$$

  where *λ*_1_ ≥ *λ*_2_ ≥ …≥*λ*_*l*_ and *λ*_*l*_ ≤ *δλ*_1_(*δ*=10^−10^).

In our opinion, it is important to combine CSP features with geometric features simultaneously by considering the consistency and complement of different features. First, discriminative information in the CSP features may be reduced using a small labelled set. However, the global distribution of geometric features may not be sufficiently reliable, because it is obtained from a small labelled set and a large unlabelled set. Therefore, we define a new comprehensive feature $\left({\hat{x}}_{i}=\left[x_{i};\;{\tilde{x}}_{i}\right]\right)$, which is a combination of the CSP feature *x*_*i*_ and geometric feature ${\tilde{x}}_{i}$ with equal weight. Both *x*_*i*_ and $\tilde{x}$ are column vectors.

#### 2.2.2. A New Balancing Constraint

To prevent all unlabelled samples from being assigned to the same class, it is assumed in LDS that the labelled and unlabelled samples have the same ratio of positive to negative samples by adding the following balancing constraint on the minimization problem in equation (1):

(8) $$\begin{array}{c} \frac{1}{U}\overset{L+U}{\underset{i=L+1}{\sum }}\left(wx_{i}+b\right)=\frac{1}{L}\overset{L}{\underset{i=1}{\sum }}y_{i}. \end{array}$$

Many TSVM methods follow this idea in LDS [20, 21, 24]. However, one problem is that the distribution of a limited labelled set cannot always represent that of a large unlabelled set, especially when the existing labelled set is unbalanced.

**Figure 2:**
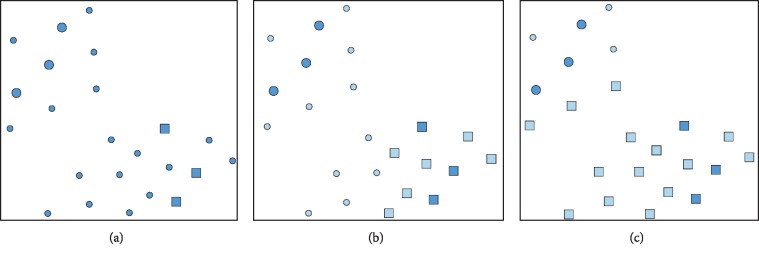
An example of binary classification. (a) A binary classification problem. (b) Classification results with one base learner. (c) Classification results with another base learner (this figure was adopted from Zhang et al. [26]).

To address this problem, Zhang trained diverse base learners based on different hypotheses regarding the distribution of positive and negative unlabelled samples; an ensemble method based on clustering evaluation means was proposed [26].  Figure 2 shows a binary classification problem and two possible classification consequences.

As illustrated in Figure 2(a), the larger solid circle and square denote labelled samples in two different classes. The extra dots are the unlabelled samples. Zhang constructed a set of base learners based on various disturbance factors that were correlated with the ratio of positive to negative unlabelled samples; the ratio ranged from 1 : 9 to 9 : 1. Figures 2(b) and 2(c) illustrate different results for the two base learners. As shown in Figure 2, neither of these two classification results is satisfactory. As a result, Zhang used *k*-means to cluster the diverse base learners and employed the clustering evaluation index to evaluate the clustering effect [26].

In general, training multiple base learners is time-consuming. Thus, we attempt to exploit a simple method that considers all possible distributions of the unlabelled set. We assume that *μ* is the average ratio of the positive samples to all samples in the unlabelled set:

(9) $$\begin{array}{c} \mu =\frac{1}{U+1}\overset{U}{\underset{i=0}{\sum }}\frac{i}{U}. \end{array}$$

The number of positive unlabelled samples varies from 0 to *U*, which could cover all cases. If each ratio is equally weighted, the value of *μ* is 0.5. Thus, the average ratio of positive to negative unlabelled data is 1 : 1, regardless of the distribution of the labelled samples. Therefore, we modify the balancing constraint using comprehensive features as follows:

(10) $$\begin{array}{c} \frac{1}{U}\overset{L+U}{\underset{i=L+1}{\sum }}\left(w{\hat{x}}_{i}+b\right)=\frac{1}{U}\overset{L+U}{\underset{i=L+1}{\sum }}y_{i}=0. \end{array}$$

#### 2.2.3. Description of ITSVM

As depicted in Figure 1, in ITSVM, we use CCCP to solve the nonconvex optimization problem under a new balancing constraint after generating the comprehensive features for all samples.

In CCCP, the cost function *J*(*θ*) given in equation (2) can be decomposed into convex and concave parts: *J*(*θ*)=*J*_convex_(*θ*)+*J*_concave_(*θ*). In addition, the concave part is approximated by its tangent ∂*J*_concave_(*θ*)/∂*θ*. CCCP employs a ramp loss function *R*_s_(*t*)=*H*_1_(*t*) − *H*_s_(*t*) for the labelled samples and a symmetric ramp loss function *SR*_s_(*t*)=*R*_s_(*t*)+*R*_s_(−*t*)for the unlabelled samples, where *H*_s_(*t*)=max(0,  *s* − *t*). It is clear that *H*_s_(*t*) is a clipped version of *H*_1_(*t*). −1 < *s* ≤ 0 is a user-defined parameter, which defines where *H*_1_(*t*) is clipped.

Each unlabelled sample is duplicated when using *SR*_s_(*t*). Each original unlabelled sample and the corresponding duplicated sample are assigned a positive or negative label, respectively, as follows:

(11) $$\begin{array}{c} \begin{array}{cc} \forall_{i=L+1}^{L+U}: & x_{i},\;y_{i}=+1;\;\\ \forall_{i=L+U+1}^{L+2U}: & x_{i}=x_{i-U},\;y_{i}=-1. \end{array} \end{array}$$

By using *R*_s_(*t*) and *SR*_s_(*t*), the convex and concave parts of *J*(*θ*) can be written as

(12) $$\begin{array}{c} J_{\text{convex}}\left(\theta \right)=\frac{1}{2}{\left\|w\right\|}^{2}+C_{1}\overset{L}{\underset{i=1}{\sum }}H_{1}\left(y_{i}\left(wx_{i}+b\right)\right)+C_{2}\overset{L+2U}{\underset{i=L+1}{\sum }}H_{1}\left(y_{i}\left(wx_{i}+b\right)\right),\\ J_{\text{concave}}\left(\theta \right)=-C_{1}\overset{L}{\underset{i=1}{\sum }}H_{s}\left(y_{i}\left(wx_{i}+b\right)\right)-C_{2}\overset{L+2U}{\underset{i=L+1}{\sum }}H_{s}\left(y_{i}\left(wx_{i}+b\right)\right). \end{array}$$

The minimization problem can be reformulated as follows by calculating the derivative of the concave part with respect to *θ*:

(13) $$\begin{array}{c} \arg\underset{\theta }{\min}\left(J\left(\theta \right)\right)=\arg\underset{\theta }{\min}\left(\frac{1}{2}{\left\|w\right\|}^{2}+C_{1}\overset{L}{\underset{i=1}{\sum }}H_{1}\left(y_{i}\left(wx_{i}+b\right)\right)+C_{2}\overset{L+2U}{\underset{i=L+1}{\sum }}H_{1}\left(y_{i}\left(wx_{i}+b\right)\right)+\overset{L+2U}{\underset{i=1}{\sum }}\beta_{i}y_{i}\left(wx_{i}+b\right)\right), \end{array}$$

where

(14) $$\begin{array}{c} \beta_{i}=\left\{\begin{array}{cc} C_{1}, & \text{ if }y_{i}\left(wx_{i}+b\right)<s\text{ and }1\leq i\leq L,\\ C_{2}, & \text{if }y_{i}\left(wx_{i}+b\right)<s\text{ and }L+1\leq i\leq L+2U,\\ 0, & \text{otherwise}. \end{array}\right. \end{array}$$

In CCCP, the balancing constraint in equation (8) can be applied to the minimization problem by introducing an extra sample (*x*_0_=(1/*U*)∑_*i*=*L*+1_^*L*+*U*^(*x*_*i*_), *y*_0_=1) and a variable (*ζ*_0_=(1/*L*) ∑_*i*=1_^*L*^ *y*_*i*_) [20, 21].

In ITSVM, the comprehensive features are used as the input of CCCP. Therefore, we replace the original features *x*_*i*_ in equations (13) and (14) with the comprehensive features ${\hat{x}}_{i}$. The new balancing constraint in equation (10) can be achieved by defining a sample $\left({\hat{x}}_{0}=\left(1/U\right)\sum_{i=L+1}^{L+U}\left({\hat{x}}_{i}\right),y_{0}=1\right)$ and a variable  (*ζ*_0_=(1/*U*)∑_*i*=*L*+1_^*L*+*U*^ *y*_*i*_=0).

ITSVM can converge quickly after at most five iterations. In the *k*th iteration, the hyperplane parameter group *θ*=(*w*, *b*) can be updated using a dual quadratic programming (QP) solver based on the generalized sequential minimal optimization (SMO) algorithm [31]. We define a linear kernel matrix *K* such that $K_{ij}=\langle {\hat{x}}_{i},{\hat{x}}_{j}\rangle ={\hat{x}}_{i}^{T}{\hat{x}}_{j}.$ The pseudocode of ITSVM can be seen in Algorithm 1. More details are shown in Appendix A.

### 2.3. IST-TSVM Algorithm

As shown in Figure 1, the CSP matrix may be the bottleneck in ITSVM when the number of labelled samples is small. Hence, we propose an IST-TSVM method that can update the CSP matrix using the expanded labelled set.

#### 2.3.1. Confidence Criterion

Generally, the base learners in self-training methods are supervised. Here, we use a semisupervised ITSVM as the base learner, which provides higher classification accuracy than supervised methods when the labelled set is small. We use the following confidence criterion to choose some unlabelled samples with high confidence to include in our labelled set, as this allows a tradeoff between the smallest distance to the class centre and the largest distance to the hyperplane. Note that the initial labelled set *D*_tr_ and the remaining unlabelled set *D*_te_ are defined in Section 2.2.1.

First, the comprehensive features ${\left\{{\hat{x}}_{i}\right\}}_{i=1}^{L+U}$ and their decision scores ${\left\{f\left({\hat{x}}_{i}\right)=w{\hat{x}}_{i}+b\right\}}_{i=1}^{L+U}$ for all samples are updated by CSP and ITSVM in each iteration, where ${\hat{x}}_{i}$ is the comprehensive feature of *e*_*i*_. Then, the positive labelled set *D*_tr+_ and the negative labelled set *D*_tr−_ are selected from the initial labelled set *D*_tr_ based on the signs of the decision scores. *D*_te+_ and *D*_te−_ are obtained from *D*_te_ in the same manner.

Second, the positive class-centre mean_+_ and the negative class-centre mean_−_ are calculated using *D*_tr+_ and *D*_tr−_, respectively:

(15) $$\begin{array}{c} {\text{mean}}_{+}=\text{mean}\left(f\left({\hat{x}}_{i}\right)\right),\;e_{i}\in D_{tr+},\;e_{i}⟶{\hat{x}}_{i},\\ {\text{mean}}_{-}=\text{mean}\left(f\left({\hat{x}}_{i}\right)\right),\;e_{i}\in D_{tr-},e_{i}⟶{\hat{x}}_{i}. \end{array}$$

Third, we define the following function $d\left({\hat{x}}_{i}\right)$ for all unlabelled sets while considering the distance to the class centre and the distance to the hyperplane simultaneously:

(16) $$\begin{array}{c} d\left({\hat{x}}_{i}\right)=\left\{\begin{array}{cc} \frac{\left|f\left({\hat{x}}_{i}\right)-{\text{mean}}_{+}\right|}{\left|f\left({\hat{x}}_{i}\right)\right|}, & e_{i}\in D_{te+},\;e_{i}⟶{\hat{x}}_{i},\\ \frac{\left|f\left({\hat{x}}_{i}\right)-{\text{mean}}_{-}\right|}{\left|f\left({\hat{x}}_{i}\right)\right|}, & e_{i}\in D_{te-},\;e_{i}⟶{\hat{x}}_{i}. \end{array}\right. \end{array}$$

The corresponding unlabelled sample is included as a labelled sample with higher confidence when $d\left({\hat{x}}_{i}\right)$ is smaller. Therefore, we, respectively, rearrange the positive unlabelled set *D*_te+_ and the negative unlabelled set *D*_te−_ according to the values of $d\left({\hat{x}}_{i}\right)$, which are sorted in the ascending order as follows:

(17) $$\begin{array}{c} \forall_{i=1}^{n_{\text{te}+}}:d\left({\hat{x}}_{i}\right)<d\left(\left.{\hat{x}}_{i+1}\right)\right)\;,\;e_{i}\in D_{\text{te}+},\;e_{i}⟶{\hat{x}}_{i},\\ \forall_{i=1}^{n_{\text{te}-}}:d\left({\hat{x}}_{i}\right)<d\left({\hat{x}}_{i+1}\right),\;\;e_{i}\in D_{\text{te}-},\;e_{i}⟶{\hat{x}}_{i}, \end{array}$$

where *n*_te+_ and *n*_te−_ are the sizes of *D*_te+_ and *D*_te−_, respectively. To maintain the distribution of the labelled set and avoid mislabelling unlabelled samples, the first *N* unlabelled samples are, respectively, selected from the two reordered sets *D*_te+_ and *D*_te−_, where *N*=0.5 × min(*n*_te+_, *n*_te−_). These 2*N* unlabelled samples with their predicted labels are used to construct the selected unlabelled set *D*_te_′, which yields the expanded labelled set ${\tilde{D}}_{\text{tr}}=D_{\text{tr}}\cup^{}D_{\text{te}}^{\prime }$.

#### 2.3.2. Description of IST-TSVM

In our proposed IST-TSVM method, we iteratively use the CSP feature extraction method and a semisupervised ITSVM classifier. We define at most five iterations. More details are presented in Algorithm 2.

In Algorithm 2, if the predicted labels of the unlabelled samples do not change or the classification error rate of the initial labelled set increases by more than 10% in the current iteration compared to the previous iteration, then the loop will be terminated in advance.

## 3. Experiments and Results

In this section, two well-known BCI competition datasets for MI are used to evaluate and compare the accuracies of our proposed approaches with SVM and classical TSVM classifiers.

- SVM is a traditional supervised learning classifier. A Gaussian kernel is often used in BCI systems for nonlinear SVM [8, 19]. Considering the computational load of the optimal kernel parameters, a linear form is chosen for SVM and the following TSVM algorithms in our study. The version of SVM used is SVM^light^ [32].
- TSVM-light is an efficient transductive learning method. This method switches the different labels of a pair of unlabelled data and solves the optimization problem in equation (2) with a dual solver during each training iteration [18].
- RTSVM is used to solve the primal optimization problem with an SG method. There are three parameters to be preset. Parameter *s* is used in the ramp loss function. Parameters *C*_1_ and *C*_2_ denote the punishment factors for labelled and unlabelled samples, respectively. We use the default selection of *s* = −0.2 and *C*_1_ = *C*_2_ = 4, as suggested by the authors [21].
- LDS is a graph-based TSVM approach. Like RTSVM, LDS performs a gradient descent on the primal formulation. The parameters for *ρ* and *δ* are empirically set to 1 and 10^−10^, respectively [25].
- CCCP minimizes the cost function in the dual space by using a dual QP solver [20]. Like RTSVM, CCCP and our algorithms contain parameters *s*, *C*_1_, and *C*_2_, which are preset to −0.2, 2, and 2, respectively.
- To compare the balancing constraint in equation (8) used by CCCP with the one in equation (10) used by ITSVM, we propose a method named as CCCP1, which is equivalent to CCCP, except that the original features are applied to the new balancing constraint as follows: (1/*U*)∑_*i*=*L*+1_^*L*+*U*^(*wx*_*i*_ +*b*)=(1/*U*)∑_*i*=*L*+1_^*L*+*U*^ *y*_*i*_. Therefore, *ζ*_0_ is set to 0 in CCCP1.

The purpose of our experiments is threefold. First, the small labelled sets are used to verify the effectiveness of SVM and all TSVM approaches. Second, the balanced and unbalanced labelled sets are used to evaluate the robustness of different classifiers. Because CCCP1 is entirely equivalent to CCCP under the condition of the balanced labelled sets, we only discuss the classification performance of CCCP1 under the condition of the unbalanced labelled sets. Third, we analyse their performance in terms of computation time.

### 3.1. EEG Datasets

- Dataset IV-a in BCI competition III: the dataset was recorded from five healthy subjects (*aa*, *al*, *av*, *aw*, and *ay*) with a total of 118 electrodes [33]. The dataset only contained data from four initial sessions without feedback. Each subject was shown visual cues for 3.5 s and performed three MI tasks: moving the left hand, right hand, or right foot. Only the latter two MI tasks were provided in the competition. For each subject, each MI task consisted of 140 trials. The presentation of target cues was interrupted by periods of random length ranging from 1.75 to 2.25 s, in which the subject could relax. The EEG signals were band-pass filtered between 0.05 and 200 Hz and downsampled from 1000 Hz to 100 Hz.
- Dataset II-a in BCI competition IV: data in this set were collected from nine subjects [34]. At the beginning of a trial, a fixation cross was shown on a black screen. Each subject then executed the desired MI tasks as directed by the visual cue in the form of an arrow pointing either to the left, right, down, or up (corresponding to moving the left hand, right hand, foot, or tongue). No feedback was provided. Twenty-two Ag/AgCl electrodes were used to record EEG signals, which were then sampled with 250 Hz and band-pass filtered between 0.5 and 100 Hz. In total, 72 trials per MI task were gathered from each subject on different days. To focus on the problem of binary classification, only MI EEG signals from the left and right hands were extracted for analysis.

### 3.2. Preprocessing

The two BCI competition datasets were preprocessed using the same methods. All raw EEG signals were band-pass filtered between 8 and 30 Hz using a fifth-order Butterworth filter. Then, the filtered signals were extracted from nonoverlapping time segments ranging from 0.5 to 2.5 s.

All classifiers in our experiments used CSP to generate their CSP features with three pairs of spatial filters. Our proposed algorithms added geometric features during classifier learning.

Data from every subject was randomly partitioned into two parts over ten repetitions. The first portion was used as the labelled set to train the classifier, while the second portion was used as the unlabelled set to verify the effectiveness of the classifier. To investigate the robustness of all algorithms with small labelled sets, we set *M* and *R* equal to the size of the labelled set and ratio of positive to negative labelled trials, respectively. We selected *M* from the set [10, 15, 20, 25, 30, 35, 40, 45, 50] and *R* from the set [1 : 4, 2 : 3, 1 :1, 3 : 2, 4 : 1].

### 3.3. Experiments with Balanced Labelled Sets

The ratio of positive to negative samples in the labelled set has a great effect on the performance of the classifier. Balanced and adequate labelled samples can provide higher classification accuracy, and vice versa, for unbalanced and sparse labelled samples.

For most semisupervised algorithms, more consideration is given to the number of labelled samples rather than the ratio of positive to negative labelled samples. In reality, both balanced and unbalanced labelled sets are common in classification problems. Therefore, we first conducted experiments with small balanced labelled sets.

#### 3.3.1. Classification Performance with Small Balanced Labelled Sets

For the two BCI competition datasets, the complete set for each subject consists of an equal number of positive and negative trials. Hence, the unlabelled set is also balanced after randomly selecting the same number of positive and negative labelled trials. First, we evaluated the recognition rates for the unlabelled sets using different classifiers learned from very small and balanced labelled sets (*M* = 10). For each subject, the classification accuracy was taken as an average from ten repetitions. Detailed results using two datasets are given in Tables 1 and 2. The highest classification performance is written in bold.

In Table 1, IST-TSVM performs better than the others. A paired *t*-test shows that the result of IST-TSVM (68.07 ± 17.62) is statistically higher than that of SVM (66.36 ± 15.53), TSVM-light (67.09 ± 16.13), RTSVM (64.12 ± 18.08), and LDS (63.27 ± 18.86) (*p* < 0.5). ITSVM provides slightly higher accuracy over CCCP. In addition, all TSVM methods are superior to SVM, except for RTSVM and LDS. Previous research with the same dataset led to the following categorization: strong: *al*; normal: *aa*, *aw*, and *ay*; weak: *av* [35]. As shown in Table 1, the accuracies of all algorithms for the strong subject (*al*) are greater than 90%. For the normal subject *ay*, IST-TSVM provides higher accuracy than the other algorithms. However, for the normal subject *aa* and the weak subject *av*, all classifiers provide poor results.

In Table 2, IST-TSVM stands out prominently on average for these nine subjects. IST-TSVM (70.22 ± 19.74) exhibits a significant improvement over RTSVM (68.47 ± 19.08) and LDS (68.14 ± 20.23) (*p*=0.005). ITSVM provides a 0.47% improvement over CCCP. All TSVM methods outperform SVM. Furthermore, according to the accuracy data in Table 2, the subjects can be categorized as follows: strong: A03, A08, and A09; normal: A01; weak: A02, A04, A05, A06, and A07. Likewise, for the strong subjects, the recognition rates of all methods remain considerably high. TSVM-light exhibits the highest performance for the normal subject (A01). Finally, all classifiers yield accuracies at the chance level for the weak subjects.

#### 3.3.2. Computation Time with Small Balanced Labelled Sets

In Table 3, we list the average computation time per subject for all TSVM classifiers in order to compare their operating speeds with small balanced labelled sets as mentioned above. The lowest computation time is highlighted in bold. The algorithms were implemented with a PC running Windows 7 Professional and Matlab R2015a. This PC contained an Intel (R) Core (TM) i3-6100 CPU @ 3.70 GHz and 8 GB RAM.

In Table 3, the time spent by CCCP is close to that by RTSVM. LDS is slower than RTSVM, while IST-TSVM requires much more time than ITSVM. TSVM-light is the most time-consuming algorithm. The following reasons may lead to the different running times. First, the framework of RTSVM is similar to that of CCCP. However, an SG method is used in RTSVM, while a dual solver is used in CCCP [20, 21]. Like RTSVM, a similar optimization strategy is pursued in LDS. However, LDS requires more time to compute the shortest paths for all pairs of samples. Based on CCCP, our proposed ITSVM spends more time calculating geometric features in a semisupervised way. By iteratively performing CSP and ITSVM, IST-TSVM exhibits higher accuracy at the cost of longer computation time. TSVM-light requires much more time because only one pair of unlabelled samples is switched to retrain the SVM in each iteration.

#### 3.3.3. Classification Performance with Varying Sizes of the Balanced Labelled Sets

**Figure 3:**
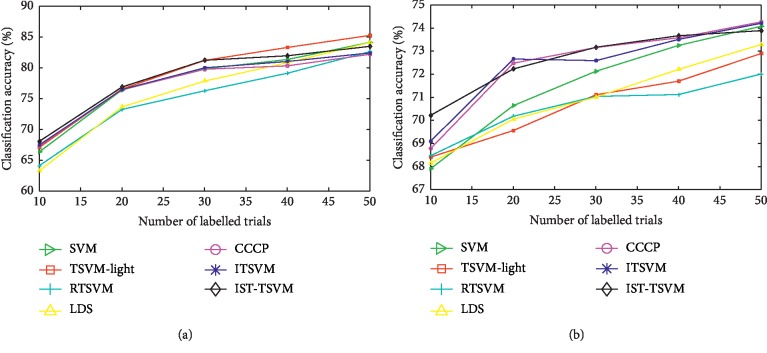
Average classification accuracy (%), with varying numbers of balanced labelled trials: (a) dataset IV-a (*R* = 1 : 1); (b) dataset II-a (*R* = 1 : 1).

We also selected balanced labelled sets with different sizes to search for more convincing results. The average classification accuracies for all subjects are plotted in Figures 3(a) and 3(b), where the numbers of labelled trials on dataset IV-a from BCI competition III and dataset II-a from BCI competition IV, respectively, are variable. The horizontal axis presents different values of *M* in intervals of ten trials.

As shown in Figure 3(a), IST-TSVM outperforms the others when the number of labelled trials is less than 30. However, TSVM-light provides high results as the number of labelled trials increases. As shown in Figure 3(b), IST-TSVM performs better than the other algorithms in most instances. The accuracy of TSVM-light is less than that of SVM. As shown in Figures 3(a) and 3(b), SVM is superior to RTSVM and LDS in terms of accuracy. In addition, ITSVM provides slightly higher recognition rates than CCCP when the number of labelled trials is less than 20. For all classifiers, the classification accuracies improve as the number of labelled trials increases if the labelled sets are balanced.

### 3.4. Experiments with Unbalanced Labelled Sets

#### 3.4.1. Classification Performance with Small Unbalanced Labelled Sets

In most datasets, the number of positive labelled samples is often equal or similar to the number of negative labelled samples. However, we do not rule out some cases. For example, the labelled set is not always balanced in the process of online training. In Tables 4 and 5, for each subject in the two datasets, we set *M* and *R* to 20 and 1 : 4, respectively. To compare different balancing constraints, the results of CCCP1 are also shown in Tables 4 and 5.

Table 4 shows that our proposed algorithms perform better than the other algorithms, even using extremely small unbalanced labelled sets. Paired *t*-test results show that IST-TSVM (63.25 ± 18.02) provides higher accuracy than that of SVM (50.82 ± 5.45), RTSVM (49.25 ± 3.01), and LDS (48.18±0.67) (*p* < 0.5). Compared to SVM, TSVM-light provides higher accuracy in most instances. For the strong subject *al*, IST-TSVM exhibits the highest accuracy. For the normal subject *aa* and the weak subject *av*, all classifiers produce results with low accuracy due to the unbalanced labelled sets and inherent characteristics of the subjects.

Similarly, one can see the advantage of IST-TSVM in Table 5. Paired *t*-test results reveal a clear difference in the accuracy of IST-TSVM (69.43 ± 20.57) and that of RTSVM (53.67 ± 10.07) (*p* < 0.05). ITSVM performs moderately better than CCCP for seven out of nine subjects. Compared to Table 2, the average accuracies for SVM, RTSVM, and LDS decrease abruptly to approximately 55%. For most subjects, TSVM-light provides relatively higher recognition rates than SVM. Regarding the strong and normal subjects, the accuracies of CCCP and our methods are very high with the small and extremely unbalanced labelled sets. All classifiers produce an accuracy near 50% for the weak subjects.

Overall, the results in Tables 4 and 5 show that IST-TSVM can be used to differentiate strong and weak subjects with extremely small unbalanced labelled sets. In addition, ITSVM provides greater accuracy than CCCP and CCCP1. For these two datasets, the accuracy of CCCP is close to that of CCCP1. CCCP performs moderately better than CCCP1 for two out of five subjects in dataset IV-a and for three out of nine subjects in dataset II-a. The average accuracy of CCCP1 is slightly lower than that of CCCP.

#### 3.4.2. Classification Performance with Varying Sizes and Distributions of Labelled Sets

We randomly selected labelled sets with different sizes and distributions (*M* ∈ [10, 15, 20, 25, 30, 35, 40, 45, 50] and *R* ∈ [1 : 4, 2 : 3, 3 : 2, 4 : 1]) from the two BCI datasets. First, to further evaluate the effect of different balancing constraints, the accuracies of CCCP and CCCP1 are shown in Tables 6 and 7.

**Figure 4:**
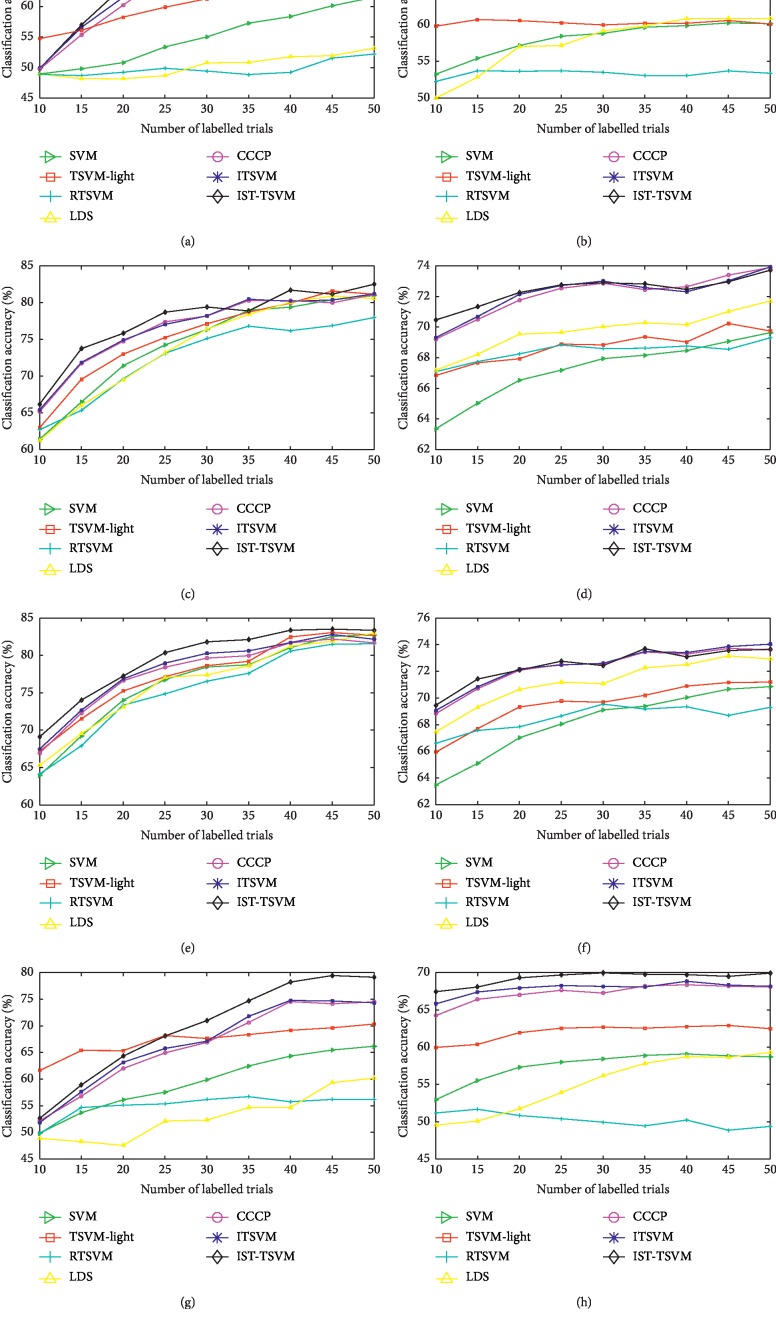
Average classification accuracy (%), with varying numbers of labelled trials (*M*) and ratios of positive to negative labelled trials (*R*) with the two datasets: (a) dataset IV-a (*R* = 1 : 4); (b) dataset II-a (*R* = 1 : 4); (c) dataset IV-a (*R* = 2 : 3); (d) dataset II-a (*R* = 2 : 3); (e) dataset IV-a (*R* = 3 : 2); (f) dataset II-a (*R* = 3 : 2); (g) dataset IV-a (*R* = 4 : 1); (h) dataset II-a (*R* = 4 : 1).

In Table 6, for each *R*, CCCP1 performs slightly better than CCCP no less than four times. In Table 7, CCCP1 provides slightly better accuracies than CCCP no less than five times when *R* is 3 : 2 or 4 : 1. In total, for each *R*, the average accuracy of CCCP1 is equal to or slightly higher than that of CCCP after averaging nine values in the corresponding column. To evaluate the performance of more algorithms, the classification accuracies of all classifiers except for CCCP1, with varying numbers of labelled trials (*M*) and ratios of positive to negative labelled trials (*R*), are plotted in Figures 4(a)–4(h).

As illustrated in Figures 4(a)–4(h), IST-TSVM shows compelling validity in most cases. The differences between ITSVM and CCCP, with the extremely unbalanced labelled sets (*R* = 1 : 4 and 4 : 1), are more apparent than those with comparatively unbalanced labelled sets (*R* = 2 : 3 and 3 : 2). The recognition rates provided by TSVM-light are always higher than those of SVM. However, RTSVM and LDS have lower accuracies than SVM with the extremely unbalanced labelled sets. In contrast to Figure 3, the performances of RTSVM with *R* = 1 : 4 and 4 : 1 are close to 50%. Nevertheless, the accuracies of RTSVM with *R* = 2 : 3 and 3 : 2 are much higher. In general, most TSVM methods are more suitable for the small unbalanced labelled sets as compared to the supervised SVM, except for RTSVM and LDS. Moreover, our algorithms are comparatively insensitive to the distribution of labelled trials.

## 4. Discussion

In this section, we discuss how various factors affect the classification performance of our proposed algorithms.

### 4.1. Impact of a Dual QP Solver

All TSVM methods mentioned above can be divided into two groups. The first group solves the primal optimization problem including RTSVM and LDS. The first group is suitable for large-scale datasets that contain millions of samples. However, in MI-based BCI systems, it is difficult to collect many samples for each subject due to the large invariability between sessions. According to the experimental results, it is clear that RTSVM and LDS cannot make full use of their merits with small-scale EEG datasets. In contrast, the second group (TSVM-light, CCCP, and our proposed algorithms) minimizes the cost function using a dual QP solver. CCCP can be used to overcome the nonconvex problem in TSVM-light. Thus, in most cases, CCCP performs better than TSVM-light. Moreover, because we use CCCP to solve the optimization problem, our algorithms and CCCP exhibit similar classification accuracies, as shown in Figures 3 and 4. Consequently, a dual QP solver plays an important role in enhancing the recognition rates of small-sized EEG sets.

### 4.2. Impact of the Comprehensive Features

In our proposed algorithms, we generate the comprehensive features for all samples by combining the CSP features with the geometric features. Under the condition of balanced labelled sets, equation (8) in CCCP is nearly equivalent to equation (10) in ITSVM, except for the use of different features. Thus, the results in Tables 1 and 2, as well as the results in Figure 3, show that the improvement provided by ITSVM compared to CCCP is attributed to the comprehensive features. Compared to CCCP, ITSVM adds the geometric features that can provide an inherent distribution of the data based on the labelled and unlabelled data. However, because the geometric features are transformed from the CSP features, they may not be sufficiently correct when the labelled set is small. Therefore, ITSVM provides a slight improvement over CCCP.

### 4.3. Impact of a New Balancing Constraint

To address the unknown distribution of the unlabelled set, we consider the various distributions of the unlabelled set and create a new balancing constraint. CCCP1 is equivalent to CCCP except for different values of *ζ*_0_. *ζ*_0_ is set to 0 to achieve the new constraint in CCCP1. However, *ζ*_0_ is set to (1/*L*)∑_*i*=1_^*L*^ *y*_*i*_ to achieve the traditional constraint in CCCP. Therefore, as shown in Tables 4 [Table tab5] [Table tab6]–7, the results of CCCP1 are close to those of CCCP. For each subject in the two BCI datasets, the number of positive samples is equal to the number of negative samples. Thus, the real ratio of positive to negative unlabelled samples will be 4 : 1, if the value of *R* is 1 : 4. However, the assumed ratio of positive to negative unlabelled samples is 1 : 4 for CCCP and 1 : 1 for CCCP1. It is clear that these two assumptions are quite different from the real distribution of the unlabelled set. As shown in Tables 6 and 7, the average accuracy of CCCP1 is equal to or slightly higher than that of CCCP under the condition of different unbalanced labelled sets. Therefore, it is feasible that we consider all possible distributions of the unlabelled set with equal weight. Moreover, following the experimental results shown in Tables 4 and 5, as well as the results in Figures 4(a), 4(b), 4(g), and 4(h), one can see that ITSVM provides higher accuracy compared to CCCP for extremely unbalanced labelled sets. We suggest that this is due to the new constraint and comprehensive features used in ITSVM.

### 4.4. Impact of the Confidence Criterion and Self-Training Model

For ITSVM, the unlabelled samples are only used in the classification phase. If the labelled set is small, the CSP transformation matrix may not be very reliable. Therefore, IST-TSVM uses the unlabelled samples from feature extraction to classifier learning. Overall, IST-TSVM exhibits its superiority using small labelled sets with balanced or unbalanced classes as depicted in Figures 3 and 4. In addition, IST-TSVM can be used to distinguish strong and weak subjects, as shown in Tables 4 and 5. We postulate that the combination of the confidence criterion and self-training model effectively improves the classification accuracy of IST-TSVM. Our confidence criterion selects the most useful unlabelled samples that are close to the class centre and far from the hyperplane simultaneously. However, if these unlabelled samples lead to convergence of the classification results for unlabelled samples or sharp degeneration of recognition rates of labelled samples, our self-training model will terminate the current iteration.

## 5. Conclusion

In summary, we introduce two improved TSVM algorithms with the goal of reducing the calibration time for BCI subjects on the premise of accurate classification in MI-based BCI systems. Our algorithms effectively incorporate a graph-based model and a self-training model into the TSVM model. To capture the inherent distribution of all samples, we use a cosine distance to measure the pairwise distance between two samples and build the nearest neighbour graph by considering the influence of labelled samples with different classes. Then, to provide different views of each sample, we combine the discriminative CSP feature with a global geometric feature embedded in the nearest neighbour graph. In addition, we replace the traditional balancing constraint with a new balancing constraint in the optimization problem to address the unknown distribution of the unlabelled set. Moreover, to make full use of unlabelled samples, we develop a confidence criterion and self-training process to iteratively retrain the CSP matrix and ITSVM classifier using the initial labelled samples and the unlabelled samples with high confidence in the IST-TSVM method. Extensive experiments show that IST-TSVM is particularly powerful and outperforms all other TSVM algorithms using small labelled sets with balanced or unbalanced classes. However, there remain opportunities for improvement. For example, there is no clear difference between ITSVM and CCCP in some cases. Thus, we will further explore the geometric characteristics of all samples in future investigations. Furthermore, in order to adapt to online MI training, we plan to develop an iterative feedback strategy with fewer unlabelled samples.

## Figures and Tables

**Algorithm 1:**
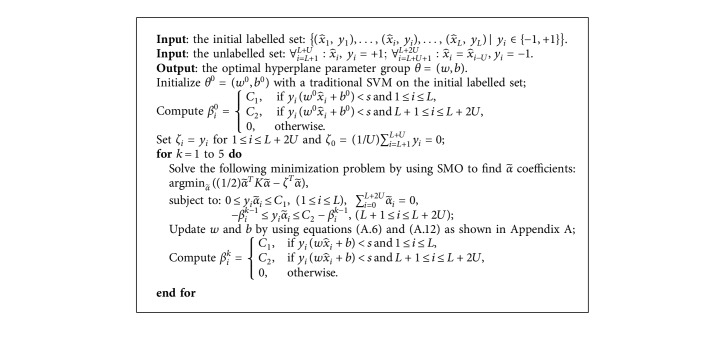
The proposed ITSVM algorithm.

**Algorithm 2:**
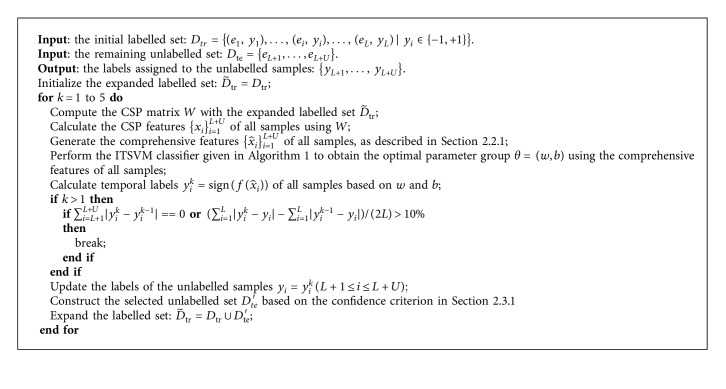
The proposed IST-TSVM algorithm.

**Table 1:** Mean accuracies with dataset IV-a (%) (*M* = 10, *R* = 1 : 1).

	SVM	TSVM-light	RTSVM	LDS	CCCP	ITSVM	IST-TSVM
*aa*	53.37	**55.44**	52.44	50.96	54.74	54.11	53.37
*al*	91.85	93.19	95.74	**96.04**	91.37	93.11	95.70
*av*	54.52	55.26	52.89	51.11	55.52	**55.56**	54.00
*aw*	64.26	59.44	57.89	56.44	**64.89**	63.44	62.96
*ay*	67.81	72.11	61.63	61.78	70.00	71.11	**74.30**
Mean	66.36	67.09	64.12	63.27	67.30	67.47	**68.07**
Std.	15.53	16.13	18.08	18.86	14.91	15.87	17.62

**Table 2:** Mean accuracies with dataset II-a (%) (*M* = 10, *R* = 1 : 1).

	SVM	TSVM-light	RTSVM	LDS	CCCP	ITSVM	IST-TSVM
A01	72.66	**80.25**	76.91	79.39	77.37	77.09	80.14
A02	50.07	50.14	49.68	49.78	50.76	51.26	**51.55**
A03	90.94	92.12	92.48	93.31	91.94	92.95	**95.54**
A04	52.55	51.62	52.41	51.98	**54.57**	54.46	53.60
A05	51.15	50.90	51.55	50.86	**52.45**	52.16	51.65
A06	56.40	55.79	56.94	53.20	**57.55**	**57.55**	56.83
A07	**57.55**	50.18	54.06	51.37	55.72	56.98	56.58
A08	89.10	91.55	90.11	90.68	89.50	89.57	**93.42**
A09	90.79	**93.09**	92.12	92.73	89.17	89.89	92.66
Mean	67.91	68.41	68.47	68.14	68.78	69.10	**70.22**
Std.	18.03	20.19	19.08	20.23	17.84	17.97	19.74

**Table 3:** Computation time comparisons (s).

	TSVM-light	RTSVM	LDS	CCCP	ITSVM	IST-TSVM
Dataset IV-a	3.89	**0.05**	0.11	0.06	0.57	2.29
Dataset II-a	15.22	0.07	0.11	**0.04**	0.59	2.56
Mean	9.56	0.06	0.11	**0.05**	0.58	2.43

**Table 4:** Mean accuracies with dataset IV-a (%) (*M* = 20, *R* = 1 : 4).

	SVM	TSVM-light	RTSVM	LDS	CCCP	CCCP1	ITSVM	IST-TSVM
*aa*	47.73	**55.62**	47.69	47.88	47.69	47.69	47.69	47.69
*al*	60.54	66.04	54.58	49.12	92.46	92.46	89.50	**93.42**
*av*	48.69	**53.42**	48.69	48.65	52.00	51.58	51.96	51.77
*aw*	48.42	59.42	47.54	47.62	54.96	55.23	62.85	**63.85**
*ay*	48.73	56.73	47.77	47.65	53.92	53.88	55.46	**59.54**
Mean	50.82	58.25	49.25	48.18	60.21	60.17	61.49	**63.25**
Std.	5.45	4.86	3.01	0.67	18.24	18.28	16.61	18.02

**Table 5 tab5:** Mean accuracies with dataset II-a (%) (*M* = 20, *R* = 1 : 4).

	SVM	TSVM-light	RTSVM	LDS	CCCP	CCCP1	ITSVM	IST-TSVM
A01	49.29	70.45	59.18	65.67	83.06	82.95	83.25	**84.40**
A02	47.76	49.18	47.99	48.81	49.44	49.59	**50.11**	**50.11**
A03	70.45	72.54	51.49	72.09	93.17	93.13	92.84	**94.93**
A04	47.76	50.97	48.21	50.78	53.06	52.72	**54.51**	54.14
A05	47.76	49.44	48.96	47.76	48.69	48.69	49.78	**50.19**
A06	48.25	**53.92**	47.65	48.02	50.34	50.41	53.21	53.43
A07	47.76	51.23	47.91	48.32	49.51	49.51	**56.34**	53.58
A08	73.92	73.43	53.06	63.99	91.87	91.90	91.90	**93.02**
A09	81.57	73.88	78.62	67.84	89.18	89.18	87.28	**91.08**
Mean	57.17	60.56	53.67	57.03	67.59	67.56	68.80	**69.43**
Std.	13.91	11.51	10.07	10.10	20.83	20.83	19.29	20.57

**Table 6 tab6:** Mean accuracies of CCCP and CCCP1 with dataset IV-a (%) (*M* ∈ [10, 15, 20, 25, 30, 35, 40, 45, 50], *R* ∈ [1 : 4, 2 : 3, 3 : 2, 4 : 1]).

*M*	*R* = 1 : 4		*R* = 2 : 3		*R* = 3 : 2		*R* = 4 : 1	
	CCCP	CCCP1	CCCP	CCCP1	CCCP	CCCP1	CCCP	CCCP1
10	49.73	**49.76**	65.15	**65.16**	**66.91**	66.88	52.17	**52.20**
15	55.36	**55.40**	**71.74**	71.67	**72.29**	72.22	56.80	**56.97**
20	**60.21**	60.17	74.74	**74.79**	76.62	**76.63**	62.00	**62.05**
25	**65.01**	**65.01**	77.39	**77.43**	**78.42**	**78.42**	**64.93**	64.91
30	67.70	**67.78**	78.18	**78.22**	**79.64**	79.61	66.90	**66.98**
35	**69.22**	69.18	80.29	**80.30**	79.96	**79.98**	70.64	**70.74**
40	**70.05**	69.74	**80.29**	80.26	81.68	**81.71**	**74.56**	74.49
45	69.75	**69.78**	**79.97**	79.91	**82.20**	82.13	**74.16**	74.07
50	70.80	**70.98**	81.10	**81.17**	81.68	**81.90**	**74.50**	**74.50**
Mean	**64.20**	**64.20**	76.54	**76.55**	77.71	**77.72**	66.30	**66.32**

**Table 7:** Mean accuracies of CCCP and CCCP1 with dataset II-a (%) (*M*  ∈  [10, 15, 20, 25, 30, 35, 40, 45, 50], *R* ∈ [1 : 4, 2 : 3, 3 : 2, 4 : 1]).

*M*	*R* = 1 : 4		*R* = 2 : 3		*R* = 3 : 2		*R* = 4 : 1	
	CCCP	CCCP1	CCCP	CCCP1	CCCP	CCCP1	CCCP	CCCP1
10	**64.98**	64.94	**69.22**	69.20	**68.82**	68.81	**64.24**	64.22
15	66.93	**67.07**	70.50	**70.53**	70.69	**70.70**	66.42	**66.58**
20	**67.59**	67.56	71.76	**71.78**	**72.08**	72.07	67.03	**67.14**
25	67.58	**67.81**	72.54	**72.56**	72.52	**72.54**	67.63	**67.72**
30	**68.14**	68.05	**72.87**	72.84	72.54	**72.55**	**67.26**	67.23
35	**67.75**	**67.75**	**72.45**	72.42	**73.45**	73.43	68.21	**68.27**
40	**67.83**	**67.83**	**72.64**	**72.64**	73.30	**73.31**	**68.37**	68.28
45	**68.08**	68.07	**73.40**	73.37	73.72	**73.74**	68.17	**68.20**
50	**68.45**	68.39	**73.89**	**73.89**	**73.63**	**73.63**	68.08	**68.10**
Mean	67.48	**67.50**	**72.14**	**72.14**	**72.31**	**72.31**	67.27	**67.30**

## Data Availability

Two datasets were employed in this study for binary classification, which are publicly available: (1) dataset IVa, BCI competition III [33]: this dataset contains EEG signals from 5 subjects, who performed 2-class MI tasks: right hand and foot. (2) dataset IIa, BCI competition IV [34]: this dataset contains EEG signals from 9 subjects, who performed 4-class MI tasks: left hand, right hand, foot, and tongue MI. In this dataset, only EEG signals from left and right hands were used. Our code and results are availableat https://github.com/xuyilu1980/tsvm.

## Appendix

### A. Derivation of the ITSVM algorithm

In ITSVM, by using the comprehensive features, the new balancing constraint, and the following definition:

(A.1) $$\begin{array}{c} H_{1}\left(t\right)=\;\max\left(0,\;1-t\right)=\min\left(\xi \right),\\ \text{subject to }:\;\xi \geq 0,\;\xi \geq 1-t, \end{array}$$

the minimization problem given in equations (13) and (14) can be rewritten as follows:

(A.2) $$\begin{array}{c} \arg\underset{\theta }{\min}\left(\frac{1}{2}{\left\|w\right\|}^{2}+C_{1}\overset{L}{\underset{i=1}{\sum }}\xi_{i}+C_{2}\overset{L+2U}{\underset{i=L+1}{\sum }}\xi_{i}+\overset{L+2U}{\underset{i=1}{\sum }}\beta_{i}y_{i}\left(w{\hat{x}}_{i}+b\right)\right)\\ \begin{array}{cc} \text{subject to}: & \frac{1}{U}\overset{L+U}{\underset{i=L+1}{\sum }}\left(w{\hat{x}}_{i}+b\right)=\frac{1}{U}\overset{L+U}{\underset{i=L+1}{\sum }}y_{i},\\ & \begin{array}{c} y_{i}\left(w{\hat{x}}_{i}+b\right)\geq 1-\xi_{i},\;1\leq i\leq L+2U,\\ \xi_{i}\geq 0,\;\;1\leq i\leq L+2U, \end{array} \end{array} \end{array}$$

where

(A.3) $$\begin{array}{c} \beta_{i}=\left\{\begin{array}{cc} C_{1}, & \text{ if }y_{i}\left(w{\hat{x}}_{i}+b\right)<s\text{ and }1\leq i\leq L,\\ C_{2}, & \text{ if }y_{i}\left(w{\hat{x}}_{i}+b\right)<s\text{ and }L+1\leq i\leq L+2U,\\ 0,\; & \text{otherwise}. \end{array}\right. \end{array}$$

We introduce the Lagrangian variables *α*_*i*_(0 ≤ *i* ≤ *L*+2*U*) and *ν*_*i*_(1 ≤ *i* ≤ *L*+2*U*) as follows:

(A.4) $$\begin{array}{c} ℒ\left(w,b,\xi ,\alpha ,\nu \right)=\frac{1}{2}{\left\|w\right\|}^{2}+C_{1}\overset{L}{\underset{i=1}{\sum }}\xi_{i}+C_{2}\overset{L+2U}{\underset{i=L+1}{\sum }}\xi_{i}+\overset{L+2U}{\underset{i=1}{\sum }}\beta_{i}y_{i}\left(w{\hat{x}}_{i}+b\right)-\alpha_{0}\left(\frac{1}{U}\overset{L+U}{\underset{i=L+1}{\sum }}\left(w{\hat{x}}_{i}+b\right)-\frac{1}{U}\overset{L+U}{\underset{i=L+1}{\sum }}y_{i}\right)-\overset{L+2U}{\underset{i=1}{\sum }}\alpha_{i}\left(y_{i}\left(w{\hat{x}}_{i}+b\right)-1+\xi_{i}\right)-\overset{L+2U}{\underset{i=1}{\sum }}\nu_{i}\xi_{i}, \end{array}$$

where *α*_0_ ≠ 0, *α*_*i*_, *ν*_*i*_ ≥ 0 for *i* ≥ 1. We compute the derivatives as follows:

(A.5) $$\begin{array}{c} \frac{\partial ℒ}{\partial w}=w-\overset{L+2U}{\underset{i=1}{\sum }}y_{i}\left(\alpha_{i}-\beta_{i}\right){\hat{x}}_{i}-\frac{\alpha_{0}}{U}\overset{L+U}{\underset{i=L+1}{\sum }}{\hat{x}}_{i},\\ \frac{\partial ℒ}{\partial b}=-\overset{L+2U}{\underset{i=1}{\sum }}y_{i}\left(\alpha_{i}-\beta_{i}\right)-\alpha_{0},\\ \frac{\partial ℒ}{\partial \xi_{i}}=C_{1}-\alpha_{i}-\nu_{i},\;1\leq i\leq L,\\ \frac{\partial ℒ}{\partial \xi_{i}}=C_{2}-\alpha_{i}-\nu_{i},\;L+1\leq i\leq L+2U.\; \end{array}$$

For simplification, an extra sample $\left({\hat{x}}_{0}=\left(1/U\right)\sum_{i=L+1}^{L+U}{\hat{x}}_{i},y_{0}=1\right)$ is defined. Setting *β*_0_ and the derivatives to zero yields

(A.6) $$\begin{array}{c} w=\overset{L+2U}{\underset{i=0}{\sum }}y_{i}\left(\alpha_{i}-\beta_{i}\right){\hat{x}}_{i},\\ \overset{L+2U}{\underset{i=0}{\sum }}y_{i}\left(\alpha_{i}-\beta_{i}\right)=0,\\ 0\leq \alpha_{i}\leq C_{1},\;1\leq i\leq L,\\ 0\leq \alpha_{i}\leq C_{2},\;L+1\leq i\leq L+2U. \end{array}$$

Then, the minimization problem in equation (A.2) can be rewritten as

(A.7) $$\begin{array}{c} \arg\underset{\alpha }{\min}\left(\frac{1}{2}\overset{L+2U}{\underset{i,j=0}{\sum }}y_{i}y_{j}\left(\alpha_{i}-\beta_{i}\right)\left(\alpha_{j}-\beta_{j}\right){\hat{x}}_{i}{}^{T}{\hat{x}}_{j}-\overset{L+2U}{\underset{i=1}{\sum }}\alpha_{i}-\alpha_{0}\frac{1}{U}\overset{L+U}{\underset{i=L+1}{\sum }}y_{i}\right),\\ \begin{array}{cc} \text{subject to }: & 0\leq \alpha_{i}\leq C_{1},\;1\leq i\leq L,\\ & 0\leq \alpha_{i}\leq C_{2},\;L+1\leq i\leq L+2U,\\ & \overset{L+2U}{\underset{i=0}{\sum }}y_{i}\left(\alpha_{i}-\beta_{i}\right)=0. \end{array} \end{array}$$

If we define *ζ*_0_=1/*U*∑_*i*=*L*+1_^*L*+*U*^ *y*_*i*_=0 and *ζ*_*i*_=*y*_*i*_ for 1 ≤ *i* ≤ *L*+2*U*, and note *K* the linear kernel matrix such that

(A.8) $$\begin{array}{c} K_{ij}=\langle {\hat{x}}_{i},{\hat{x}}_{j}\rangle ={\hat{x}}_{i}{}^{T}{\hat{x}}_{j}, \end{array}$$

and perform

(A.9) $$\begin{array}{c} {\tilde{\alpha }}_{i}=y_{i}\left(\alpha_{i}-\beta_{i}\right). \end{array}$$

The minimization problem in equation (A.7) can be rewritten as follows:

(A.10) $$\begin{array}{c} \arg\underset{\tilde{\alpha }}{\min}\left(\frac{1}{2}{\tilde{\alpha }}^{T}K\tilde{\alpha }-\zeta^{T}\tilde{\alpha }\right)\\ \begin{array}{cc} \text{subject to}: & 0\leq y_{i}{\tilde{\alpha }}_{i}\leq C_{1},\;1\leq i\leq L,\\ & -\beta_{i}\leq y_{i}{\tilde{\alpha }}_{i}\leq C_{2}-\beta_{i},\;\;\;L+1\leq i\leq L+2U,\\ & \overset{L+2U}{\underset{i=0}{\sum }}{\tilde{\alpha }}_{i}=0. \end{array}\; \end{array}$$

We can extend the method to the nonlinear case by defining the kernel matrix *K* as follows:

(A.11) $$\begin{array}{c} K_{ij}=\langle \Phi \left({\hat{x}}_{i}\right),\Phi \left({\hat{x}}_{j}\right)\rangle . \end{array}$$

For simplification, we only consider the linear case. In order to obtain the optimal hyperplane parameter group *θ*=(*w*, *b*), five iterations are executed in Algorithm 1. In each iteration, the bounds in equation (A.10) on the ${\tilde{\alpha }}_{i}$ are adjusted after each update of *β* and the ${\tilde{\alpha }}_{i}$ coefficients are found by SMO. Then, the hyperplane normal *w* can be updated by using equation (A.6). The hyperplane bias *b* can be obtained by using the following constraints:

(A.12) $$\begin{array}{c} 0<\alpha_{i}<C_{1},\;1\leq i\leq L⟶y_{i}\left(w{\hat{x}}_{i}+b\right)=1,\\ 0<\alpha_{i}<C_{2},\;L+1\leq i\leq L+2U⟶y_{i}\left(w{\hat{x}}_{i}+b\right)=1. \end{array}$$
